# Costs of implementing a multi-site facilitation intervention to increase access to medication treatment for opioid use disorder

**DOI:** 10.1186/s43058-023-00482-8

**Published:** 2023-08-10

**Authors:** Carla C. Garcia, Mark Bounthavong, Adam J. Gordon, Allison M. Gustavson, Marie E. Kenny, Wendy Miller, Aryan Esmaeili, Princess E. Ackland, Barbara A. Clothier, Ann Bangerter, Siamak Noorbaloochi, Alex H. S. Harris, Hildi J. Hagedorn

**Affiliations:** 1 Health Economics Resource Center, VA Palo Alto Health Care System, Palo Alto, CA USA; 2 UCSD Skaggs School of Pharmacy & Pharmaceutical Sciences, San Diego, CA USA; 3 Vulnerable Veteran Innovative PACT (VIP) Initiative, Informatics, Decision-Enhancement, and Analytic Sciences Center (IDEAS, Salt Lake City Veterans Affairs Health Care System, Salt Lake City, UT USA; 4 Program for Addiction Research, Clinical Care, Knowledge and Advocacy (PARCKA), Department of Internal Medicine, University of Utah School of Medicine, Salt Lake City, UT USA; 5 Center for Care Delivery & Outcomes Research, Minneapolis Veterans Affairs Health Care System, Minneapolis, MN USA; 6 Department of Psychiatry, School of Medicine, University of Minnesota, Minneapolis, MN USA; 7 Center for Innovation to Implementation, VA Palo Alto Health Care System, Palo Alto, CA USA; 8 Department of Surgery, School of Medicine, Stanford University, Stanford, CA USA

**Keywords:** External facilitation, Cost analysis, Implementation science, Medication for opioid use disorder, Evidence-based practice

## Abstract

**Background:**

The United States has been grappling with the opioid epidemic, which has resulted in over 75,000 opioid-related deaths between April 2020 and 2021. Evidence-based pharmaceutical interventions (buprenorphine, methadone, and naltrexone) are available to reduce opioid-related overdoses and deaths. However, adoption of these medications for opioid use disorder has been stifled due to individual- and system-level barriers. External facilitation is an evidence-based implementation intervention that has been used to increase access to medication for opioid use disorder (MOUD), but the implementation costs of external facilitation have not been assessed. We sought to measure the facility-level direct costs of implementing an external facilitation intervention for MOUD to provide decision makers with estimates of the resources needed to implement this evidence-based program.

**Methods:**

We performed a cost analysis of the pre-implementation and implementation phases, including an itemization of external facilitation team and local site labor costs. We used labor estimates from the Bureau of Labor and Statistics, and sensitivity analyses were performed using labor estimates from the Veterans Health Administration (VHA) Financial Management System general ledger data.

**Results:**

The average total costs for implementing an external facilitation intervention for MOUD per site was $18,847 (SD 6717) and ranged between $11,320 and $31,592. This translates to approximately $48 per patient with OUD. Sites with more encounters and participants with higher salaries in attendance had higher costs. This was driven mostly by the labor involved in planning and implementation activities. The average total cost of the pre-implementation and implementation activities were $1031 and $17,816 per site, respectively. In the sensitivity analysis, costs for VHA were higher than BLS estimates likely due to higher wages.

**Conclusions:**

Implementing external facilitation to increase MOUD prescribing may be affordable depending on the payer’s budget constraints. Our study reported that there were variations in the time invested at each phase of implementation and the number and type of participants involved with implementing an external facilitation intervention. Participant composition played an important role in total implementation costs, and decision makers will need to identify the most efficient and optimal number of stakeholders to involve in their implementation plans.

**Supplementary Information:**

The online version contains supplementary material available at 10.1186/s43058-023-00482-8.

Contributions to the literature

- Costs analyses focus on the costs of the clinical intervention and do not always describe the costs associated with implementing an evidence-based intervention. Understanding the costs of implementing an intervention can inform policymakers when constrained by a budget.
- This is the first cost analysis to measure the facility-level direct costs of implementing an external facilitation intervention for medication treatment for opioid use disorder.
- Although we found variations in the time invested at each phase of implementation and the number and type of participants involved with implementing an external facilitation intervention, decision makers may use these results to determine whether the intervention is affordable based on their budget constraint.

## Introduction

Over the last two decades, the United States (U.S.) has been grappling with the opioid epidemic, which has been further exacerbated by the coronavirus disease 2019 (COVID-19) [1]. From April 2020 to April 2021, there were 75,673 opioid-related deaths reported, an increase of 35% from the prior year [2, 3]. Evidence-based interventions to reduce opioid-related overdoses and deaths are available, such as medication treatment for opioid use disorder (MOUD) [4–10]. MOUD, which includes formulations of methadone, buprenorphine, and naltrexone, are evidence-based pharmaceutical therapies that are effective at reducing illicit use and improving patient outcomes associated with opioid use disorder (OUD) [10–22]. However, access to MOUD has been limited due to stigma, federal laws requiring providers to acquire additional specialized training, and concerns about time allocation among others [23–26].

The Veterans Health Administration (VHA), which is a part of the U.S. Department of Veterans Affairs (VA), has made progress to improve access to and prescribing of MOUD through the implementation of innovative programs [27]. One such intervention is Advancing Pharmacological Treatments for Opioid Use Disorder (ADaPT-OUD), an implementation study that was designed to examine an external facilitation intervention to reduce barriers to the provision of MOUD [28]. External facilitation is an evidence-based implementation intervention and differs from national, top-down VHA efforts by empowering local clinical teams, building on local resources and strengths, tailoring strategies to specific facility-level barriers, and promoting sustained attention to implementation [29–32]. Recent studies have reported improvements in MOUD prescribing through ADaPT-OUD [33, 34]. Gustavson and colleagues reported that seven out of eight VHA facilities that participated in ADaPT-OUD had improvements in the proportion of patients with OUD who received MOUD at the 6-month follow-up [34]. Hagedorn and colleagues reported that VHA facilities that engaged in external facilitation through ADaPT-OUD had significantly increased the proportion of patients with OUD who received MOUD from 18% at baseline to 30% at the 12-month follow-up [33]. Based in part on the success of this trial, the VHA implemented an initiative using an external facilitation intervention to improve access to MOUD in primary care, mental health, and pain clinical environments nationwide [35–37].

Having established the effectiveness of external facilitation in this setting, it is critical to perform an accompanying cost analysis to inform stakeholders who are considering implementing this evidence-based intervention [38]. Estimating the cost of the actual implementation piece of the intervention is a necessary but often overlooked exercise when performing a cost analysis of an evidence-based intervention. Without proper understanding of the costs associated with implementing an evidence-based intervention, decision makers will face uncertainty and may decline to invest in the strategy. Hence, it is necessary to itemize the costs of implementing an external facilitation intervention so that decision makers are informed about its impact on their budget. Studies on the cost of implementing evidence-based programs are increasing in the literature, thereby providing stakeholders with some estimates of the resources needed to properly implement evidence-based programs [38–41]. However, to date, there have been no formal evaluations on the cost of implementing an external facilitation strategy for MOUD. To address this issue, we performed a cost analysis of implementing an external facilitation program to increase MOUD in VHA.

## Methods

### Design

**Fig. 1:**
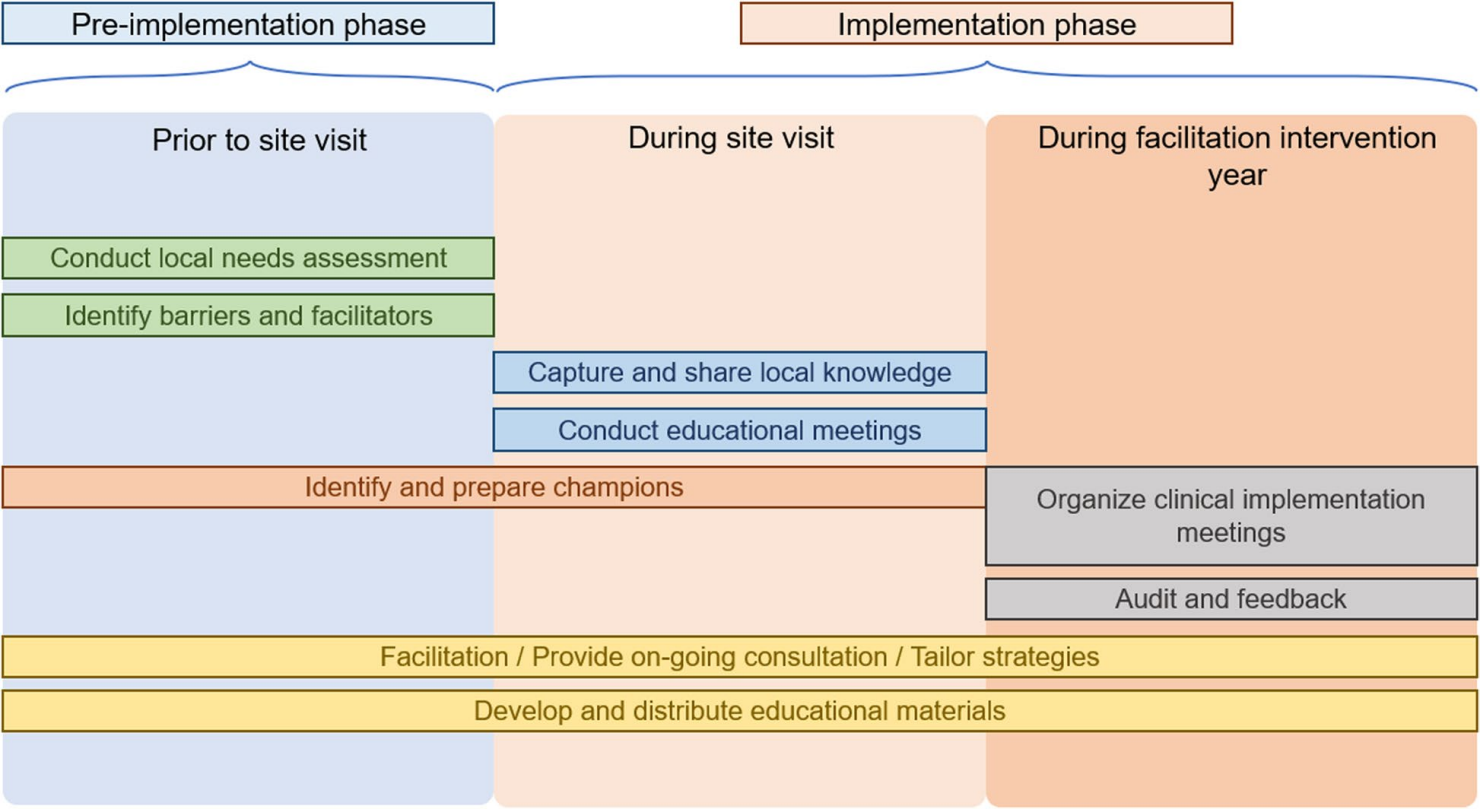
Implementation of external facilitation for ADaPT-OUD

A cost analysis using micro-costing methods was performed to assess the cost of implementing ADaPT-OUD at eight VA sites [42]. Figure 1 illustrates the implementation phases for the ADaPT-OUD intervention with additional details provided in a previous publication [28]. Costs were captured for activities during the pre-site visit, on-site visit, and facilitation that took place after the on-site visit.

### External facilitation team (EF)

The external facilitation (EF) team collaborated with internal implementation teams from the 8 sites that participated in ADaPT-OUD. The EF team consisted of 8 members: 4 psychologists, 3 research associates, and 1 physician-scientist.

### Local staff (LS)

Local non-research staff consisted of 2 groups: internal implementation teams (IIT) and other training participants (OTP).

#### Internal implementation teams (IIT)

The IIT was a small group consisting of key stakeholders (e.g., facility leaders and clinicians across specialty substance use disorder [SUD] and general health care environments) who were actively involved in leading local facilitation efforts, joined cross-site and monthly facilitation calls, and were interviewed as part of the pre-implementation needs assessment. IIT members attended didactic trainings, participated in monthly coaching calls, and completed any implementation activities during their regularly paid clinical time.

#### Other training participants (OTP)

The OTP was a large group composed of LS who attended didactic trainings at their sites. They were a more passive audience compared to IIT.

### Pre-implementation phase

The pre-implementation or planning phase was defined as the date from when the first labor-driven activity occurred (i.e., preparing resources for site visits, pre-site visit interviews) until the day before the on-site visit which varied across the eight sites. Pre-implementation activities included conducting local needs assessment for each site by interviewing local leaders and clinicians from substance use disorder clinics and other clinic settings (e.g., primary care, general mental health, pain management) that may be involved in increasing access to MOUD. Rapid qualitative analysis was performed to identify facilitators and barriers to prescribing MOUD, and a facility summary report was prepared which was discussed with the local facilitation team during the on-site visit [34, 43, 44]. The external facilitators shared a list of potential topics for face-to-face education (e.g., X-waiver training, treating OUD with buprenorphine and naltrexone, setting up a buprenorphine clinic) with each site. The IIT then selected trainings for their upcoming visit. Other pre-implementation activities by external facilitators included preparing resource materials for on-site trainings and drafting facility-specific action plans.

### Implementation phase

We defined the implementation phase as the first day of the on-site visit until the last labor-driven activity (e.g., cross-site collaborative calls, post-site visit interviews) at each site. The EF team shared the facility summary report with the IIT during the on-site facility visits, which lasted between one and two days. Goals and strategies were identified and developed, and MOUD-related educational resources were provided and targeted at the broader clinical audience. Each facility was offered buprenorphine X-waiver certification education by the EF team and 7 of 8 facilities elected to receive this training [45]. X-wavier trainings were credentialed by the American Academy of Addiction Psychiatry and consisted of “half and half” trainings: 4 h of face-to-face case studies, discussions, and lectures led by the on-site external facilitator followed by 4 h of structured web-based curricular content completed at a separate time without the facilitator [46, 47]. Aside from X-waiver trainings, all other trainings (e.g., Introduction to Provider Education and Patient Tracking Resources, Site Report Review, Action Planning) ranged in length from 0.5 to 1.75 h. While training agendas were tailored to individual sites, some aspects of the site visit agenda were standardized. The 2 lead external facilitators, in collaboration with the IIT, developed action plans for each site. These plans provided the guiding goals and action steps for the remainder of the implementation phase. Facility visits were followed by continued external facilitation, including 12 monthly coaching calls, quarterly cross-site community of practice meetings, quarterly site-level feedback reports including key metrics (e.g., MOUD/OUD ratio, number of waivered prescribers, number of patients prescribed buprenorphine in the last six months, and number of patients with a diagnosis of OUD not receiving MOUD), and quarterly newsletters.

### Data collection

For the pre-implementation and implementations phases, data collection for each site was done by the EF team to account for time spent on implementation activities by IITs, OTPs, and external facilitators. Sign-in sheets were collected at on-site trainings to capture the number of unique attendees along with the date and record of the training time, and names and occupations of OTPs. The three external facilitators who were most involved, self-reported the time spent on activities with each VA site using tracking logs. Tracking forms and definitions were adapted from a VHA-based implementation facilitation guide [48]. These tracking logs recorded the activities of implementation and the amount of time spent on each activity. Adhering to the facilitation guide, activities less than 15 min were not tracked. All data were entered into Excel spreadsheets and categorized according to activity type (e.g., preparation time, one-on-one or group interactions), communication mode (e.g., phone, email, video), and facilitation activities (e.g., assessment, planning, stakeholder engagement, coaching calls, individual consultation, education, program marketing, problem identification, and problem-solving) [48]. Administrative data were collected for pre and post-implementation interviews, including interviewee roles, dates, and duration of interviews. Finally, records from monthly facilitation and cross-site calls were collected and categorized by date, attendee occupation, and estimated length of calls (recorded in 0.25-h increments).

### Costs data

Costs of external facilitation activities were categorized into two phases: pre-implementation (or planning) and implementation. Labor costs were estimated based on the wage rate and hours invested in implementation. The wage tables came from two sources: (1) Bureau of Labor and Statistics (BLS) and (2) VHA Financial Management System (FMS) general ledger data were used. Wages based on the BLS provided a national average that would be more generalizable to non-VHA institutions. VHA FMS general ledger contains data on VHA labor and includes the number of hours worked, the average cost per hour of work, and the occupation type (using the Budget Occupation Code) [49, 50]. BLS data did not include fringe benefits; hence, we performed additional analyses by adding a 30% fringe benefit to the wage rates based on budget guidance from VA [51]. The VHA FMS data included fringe, so we calculated the hourly rate to determine unadjusted costs. Both wage rates were used to calculate the costs of LS (i.e., IIT and OTP) and EF teams.

EF teams traveled to sites to conduct face-to-face facilitation, and travel costs were captured as part of the total costs and were collected from the VHA’s travel and expense management system. External facilitators’ travel itinerary and receipts were used to group travel costs into two categories: lodging and incidentals (e.g., hotel taxes, lodging, meals, tips, and baggage fees), and transportation (e.g., airline flight, gasoline, public transportation, car rental, taxi, and app-based ride hailing).

### Analysis

Records containing information from sign-in sheets at on-site visits, interview logs, call attendance sheets, and external facilitators’ tracking reports were merged into an Excel file. These were categorized into various activities (e.g., calls, trainings, and interviews), which were further divided into subcategories (e.g., monthly facilitation calls, cross-site collaborative calls, X-waiver trainings, and pre-implementation interviews). We rounded the time spent on each activity to the nearest quarter hour. Each participant identified themselves according to their occupation or role on the sign-in sheets from the trainings. In the base-case analysis, wages for these occupations were derived from the 2020 BLS national database. Hourly wages were calculated using a formula (annual salary divided by 2080 h). Wage rates were categorized into the following: < $30 per hour, $30 to < $60 per hour, $60 to < $90 per hour, and $90 or greater per hour. Labor costs were totaled by multiplying the hourly wage of each employee by the time spent on an activity. Additional personnel labor costs were adjusted by adding 30% for fringe benefits. We presented the total costs of planning and implementation and the averaged total costs by sites including standard deviations (SD). We estimated the costs per patient (data on the number of patients with OUD at each site was based on our previous clustered, randomized-controlled trial) [33], costs per participant, and costs per encounter. We also estimated the total hours dedicated to planning and implementation and the average total hours by site including the standard deviations. A sensitivity analysis was conducted using the VHA FMS salary database to compare how total costs differed from the BLS.

## Results

### Overall summary (base-case analysis)

In the base-case, the average total costs for implementation-related activities per site was $18,847 (SD 6717) and ranged between $11,320 and $31,592 (Table 1 and Additional file 1: Table S1). The average total costs for a patient with OUD was $48 across all sites (minimum: $19, maximum: $173 per patient). For LS, a majority of the costs were due to implementation training activities [average cost per site = $11,456 (SD 7199)] followed by post-site visit activities (e.g., post-implementation interviews, cross-site collaborative calls) [average cost per site = $3052 (SD 1475)] and pre-implementation activities (e.g., pre-implementation interviews, site visit planning) [average cost per site = $396 (SD 67)] (Table 2). The average number of hours per site dedicated from LS to attend trainings was 145 h (SD 83), which was followed by post-site visit activities [average number of hours = 41 h (SD 18)] and pre-implementation activities [average number of hours = 5 h (SD 1)]. The average number of unique attendees per site at meetings, interviews, and trainings were 13, 14, and 28 attendees, respectively (Table 3).

**Table 1:** Total costs of implementation by site using labor wage data from Bureau of Labor and Statistics (BLS) and Veterans Health Administration Financial Management System (VHA FMS)^a^

Site	Number of eligible patients	BLS			VHA FMS		
		Total costs	Total costs + 30% fringe benefits	Total costs per patient + 30% fringe benefits	Total costs	Total costs + 30% fringe benefits	Total costs per patient + 30% fringe benefits
1	141	$17,786	$23,122	$164	$22,117	$28,752	$204
2	178	$11,320	$14,716	$83	$13,759	$17,887	$100
3	238	$31,592	$41,069	$173	$39,880	$51,844	$218
4	629	$21,009	$27,312	$43	$25,986	$33,782	$54
5	647	$18,316	$23,811	$37	$22,650	$29,445	$46
6	955	$14,002	$18,202	$19	$17,482	$22,727	$24
7	871	$24,151	$31,397	$36	$30,258	$39,335	$45
8	411	$12,600	$16,381	$40	$15,671	$20,372	$50
Total	4070	$150,776	$196,009		$187,803	$244,144	
Average total per site	508.75	$18,847	$24,501	$48	$23,475	$30,518	$60

^a^Does not include travel costs; includes local staff and external facilitation teams

**Table 2:** Costs of local staff by site categorized by activities^a^^,b^

Site	Pre-implementation/planning		Implementation: site trainings		Implementation: post-site visit		Total activities	
	Cost	Cost + 30% fringe benefits	Cost	Cost + 30% fringe benefits	Cost	Cost + 30% fringe benefits	Cost	Cost + 30% fringe benefits
1	$511	$664	$6038	$7850	$6076	$7899	$12,625	$16,413
2	$321	$417	$2594	$3372	$3541	$4603	$6456	$8392
3	$425	$552	$25,064	$32,583	$2826	$3673	$28,315	$36,808
4	$409	$531	$14,340	$18,642	$2067	$2687	$16,816	$21,860
5	$303	$394	$10,789	$14,026	$3821	$4968	$14,913	$19,388
6	$397	$516	$8782	$11,417	$1431	$1861	$10,610	$13,794
7	$438	$569	$17,178	$22,332	$2853	$3708	$20,469	$26,609
8	$368	$478	$6864	$8923	$1804	$2345	$9036	$11,746
Total costs	$3172	$4121	$91,649	$119,145	$24,419	$31,744	$119,240	$155,010
Average total costs per site (SD)	$396 (67)	$515 (87)	$11,456 (7199)	$14,893 (9359)	$3052 (1475)	$3968 (1917)	$14,905 (7009)	$19,376 (9112)

^a^Does not include external facilitators

^b^Includes encounters and are not unique attendees

**Table 3:** Number of participants by site stratified by position, wage category, and implementation activities

Variable	Site 1	Site 2	Site 3	Site 4	Site 5	Site 6	Site 7	Site 8	Total	Average Per Site
Total *N* (unique)	26	24	57	42	33	27	60	26	295	37
Total *N* (encounters)	253	207	299	264	259	205	268	167	1922	240
Positions (unique), *n* (%)										
Local staff										
Physicians^a^	5 (19%)	3 (13%)	23 (40%)	5 (12%)	4 (12%)	7 (26%)	25 (42%)	3 (12%)	75	9
Nurses	2 (8%)	2 (8%)	5 (9%)	4 (10%)	2 (6%)	2 (7%)	11 (18%)	3 (12%)	31	4
Nurse practitioners	2 (8%)	2 (8%)	7 (12%)	8 (19%)	4 (12%)	3 (11%)	4 (7%)	2 (8%)	32	4
Physician assistants	0 (%)	0 (%)	1 (2%)	2 (5%)	3 (9%)	0 (%)	2 (3%)	0 (%)	8	1
Psychologists^b^	1 (4%)	1 (4%)	1 (2%)	5 (12%)	2 (6%)	0 (%)	0 (%)	3 (12%)	13	2
Social workers	1 (4%)	1 (4%)	0 (%)	1 (2%)	2 (6%)	2 (7%)	0 (%)	1 (4%)	8	1
Other healthcare professions^c^	1 (4%)	4 (17%)	8 (14%)	1 (2%)	5 (15%)	3 (11%)	6 (10%)	3 (12%)	31	4
Service chiefs	8 (31%)	5 (21%)	7 (12%)	1 (2%)	6 (18%)	4 (15%)	6 (10%)	6 (23%)	43	5
External facilitators	6 (23%)	6 (25%)	5 (9%)	6 (14%)	5 (15%)	6 (22%)	6 (10%)	5 (19%)	45	6
Wages (unique), *n* (%)										
$0 < $30 per hour	3 (12%)	5 (21%)	2 (3%)	4 (10%)	4 (12%)	5 (19%)	3 (5%)	3 (12%)	29	4
$30–$60 per hour	9 (35%)	7 (29%)	16 (28%)	25 (60%)	14 (42%)	7 (26%)	19 (32%)	10 (38%)	107	13
$60–$90 per hour	0 (%)	4 (17%)	8 (14%)	2 (5%)	4 (12%)	3 (11%)	6 (10%)	3 (12%)	30	4
$90 + per hour	14 (54%)	8 (38%)	31 (54%)	11 (26%)	11 (33%)	12 (44%)	32 (53%)	10 (38%)	129	16
Attendees (uniques) at planning and implementation activities^d^, *n* (%)										
Meetings	18 (30%)	17 (41%)	9 (13%)	11 (17%)	14 (27%)	13 (30%)	13 (16%)	11 (26%)	103	13
Trainings	17 (34%)	10 (41%)	47 (67%)	36 (55%)	26 (50%)	17 (40%)	52 (67%)	17 (43%)	222	28
Interviews	18 (36%)	14 (34%)	14 (20%)	18 (28%)	12 (23%)	13 (30%)	13 (16%)	12 (30%)	114	14

^a^Includes psychiatrists

^b^Includes licensed addiction therapists

^c^Other: mental health program analyst, pharmacist, mental health coordinator, health systems specialist

^d^Includes external facilitators

Overall, most of the participants were physicians followed by EF members, service chiefs, nurse practitioners, nurses, other healthcare professions, psychologists, physician assistants, and social workers (Table 3). Additionally, a majority of participants had a wage rate of $90 or greater per hour (average number per site = 16) followed by a wage rate of $30 to $60 per hour (average number per site = 13), $60 to $90 per hour (average number per site = 4), and less than $30 per hour (average number per site = 4).

Sites with more encounters and participants with higher salaries (e.g., physicians, chiefs of staff) in attendance had higher costs. This was driven mostly by the labor involved in planning and implementation activities. For instance, Site 3 had 299 encounters with 57 unique participants; a large proportion of participants were physicians (40%) with wage rates that were $90 per hour or more (54%) (Table 1). This combination resulted in a total site-specific cost of $31,592. In contrast, Site 2 had 207 encounters with 24 unique participants that included mostly service chiefs (21%) and other healthcare professionals (17%) with wage rates of $90 or more per hour (38%) and $30 to $60 per hour (29%), respectively (Table 3). This resulted in a total site-specific cost of $11,320 (Tables 1 and 4). The average total costs per participant and per encounter were $664 and $102, respectively, across all sites (see Additional file 1: Table S1).

**Table 4:** Costs of external facilitators (EF) team, internal implementation team (IIT), and local staff (LS)^a^

	**Planning phase**									**Implementation phase**									**Total**		
	**Hours**			**Cost**			**Cost + 30% fringe benefits**			**Hours**			**Cost**			**Cost + 30% fringe benefits**			**Total hours**	**Total cost**	**Total cost + 30% fringe benefits**
	**EF hours**	**IIT hours**	**EF + IIT total hours**	**EF cost**	**IIT cost**	**EF + IIT cost**	**EF cost + 30% fringe benefits**	**IIT cost + 30% fringe benefits**	**EF + IIT total cost + 30% fringe benefits**	**EF hours**	**Local staff**	**EF + LS total hours**	**EF cost**	**Local staff**	**EF + LS cost**	**EF cost + 30% fringe benefits**	**Local staff cost times**	**EF + LS total cost + 30% fringe benefits**			
Site 1^b^	17	5	23	$854	$511	$1365	$1111	$664	$1775	83	142.75	225	$4307	$12,114	$16,421	$5599	$15,749	$21,347	248	$17,786	$23,122
Site 2^b^	16	4	20	$711	$321	$1032	$925	$417	$1342	87	95	182	$4153	$6134	$10,288	$5399	$7975	$13,374	202	$11,320	$14,716
Site 3	8	5	13	$312	$425	$737	$406	$552	$958	65	315	380	$2965	$27,890	$30,855	$3855	$36,257	$40,111	393	$31,592	$41,069
Site 4	11	6	17	$562	$409	$971	$731	$531	$1262	82	241	322	$3631	$16,407	$20,038	$4720	$21,329	$26,049	339	$21,009	$27,312
Site 5	15	4	18	$878	$303	$1181	$1142	$394	$1535	66	207	272	$2524	$14,611	$17,135	$3281	$18,994	$22,276	291	$18,316	$23,811
Site 6	12	5	17	$553	$397	$950	$719	$516	$1235	62	145	207	$2838	$10,214	$13,052	$3690	$13,278	$16,967	224	$14,002	$18,202
Site 7	12	4	17	$702	$438	$1139	$912	$569	$1481	74	235	309	$2981	$20,031	$23,012	$3875	$26,040	$29,915	326	$24,151	$31,397
Site 8	9	5	15	$503	$368	$870	$654	$478	$1132	71	113	183	$3062	$8668	$11,730	$3981	$11,268	$15,249	198	$12,601	$16,381
**Totals**	**100**	**39**	**138**	**$5077**	**$3170**	**$8246**	**$6599**	**$4121**	**$10,720**	**589**	**1493**	**2081**	**$26,462**	**$116,068**	**$142,530**	**$34,400**	**$150,889**	**$185,289**	**2220**	**$150,776**	**$196,009**

^a^Local staff is comprised of IIT and other training participants (OTP)

^b^Sites 1 and 2 had consecutive site visit dates so some administrative time and costs were split among these sites

Overall, more time and greater costs occurred in the implementation phase compared to the planning phase across all sites. External facilitators accrued 589 total hours in the implementation phase across all sites resulting in a cost of $26,462. In the planning phase, EF accrued 100 h resulting in a cost of $5077. Similarly, LS at each of the 8 sites had greater efforts in the implementation phase with 1493 total hours across all sites resulting in an implementation phase cost of $116,068 in contrast to the pre-implementation phase where LS contributed 39 total hours across sites resulting in a cost of $3170 (Table 4).

### Pre-implementation/planning phase

**Fig. 2:**
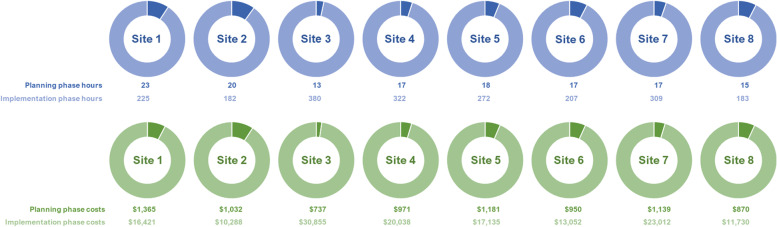
Hours and costs of facilitation phases (planning and implementation) by site

The average total cost of the pre-implementation phase activities was $1031 per site and ranged between $737 and $1365 (Table 4, Fig. 2). The average time invested in pre-implementation activities was 17 h per site and ranged between 13 (Site 3) and 23 h (Site 1) (Fig. 2). As the number of hours increased, the total costs also increased (see Additional file 1: Fig. S1). These costs were based on the EF team’s time conducting local needs assessment and the time dedicated by IITs. EF and IIT engaged in more pre-implementation activities at Site 1 (17 and 5 h, respectively) versus Site 3 (8 and 5 h, respectively). More time was spent on sites 1 and 2 (17 and 16 h, respectively) by the EF team as more time was expended on developing materials, which were then used for the remaining sites (see Table 4). Overall, the EF team had more variability in hours and costs across sites ranging from 8 to 17 h and $312 to $878 in costs than IIT (4 to 6 h, $303 to $511). Two external facilitators (A and B) engaged in more planning activities than other external facilitators (see Additional file 1: Table S2) because they were the only team members who traveled to sites where they led trainings with OTPs and met with IITs.

The average number of pre-implementation interviews was 9 per site and ranged from 7 to 11 interviews (Table 5). The average total cost per site of pre-implementation interviews for IITs was $396 (SD 67) and ranged from $303 to $511. The average time spent by LS for interviews per site was 5 h (SD 1) and ranged from 4 to 6 h. More than half of the EF team conducted pre-implementation interviews (see Additional file 1: Table S2). Differences in the site-specific costs for interviews were attributed to the occupation of interviewees and interviewers rather than the number or duration of interviews.

**Table 5:** Total encounters of local staff by site stratified by meetings, site visits/training, and interviews

Variable	Site 1	Site 2	Site 3	Site 4	Site 5	Site 6	Site 7	Site 8	**Totals**
**Meetings**									
Monthly facilitation calls	63 (90%)	54 (84%)	23 (88%)	21 (91%)	48 (92%)	26 (93%)	31 (94%)	20 (95%)	**286**
Cross-site collaboration calls	7 (10%)	10 (16%)	3 (12%)	2 (9%)	4 (8%)	2 (7%)	2 (6%)	1 (5%)	**31**
**Meetings total**	**70**	**64**	**26**	**23**	**52**	**28**	**33**	**21**	
**Site visits/trainings**									
Advanced topics for waivered providers and difficult cases	4 (8%)	4 (16%)	22 (12%)	n/a	10 (9%)	13 (18%)	16 (12%)	9 (16%)	**74**
Buprenorphine and naltrexone for the treatment of opioid use disorder (OUD)^a^	n/a	n/a	n/a	12 (10%)	18 (16%)	12 (16%)	14 (10%)	11 (20%)	**67**
Models for integrating OUD treatment into primary care and general mental health settings^a^	n/a	n/a	24 (13%)	12 (10%)	18 (16%)	12 (16%)	20 (14%)	11 (20%)	**97**
National efforts toward stepped care for OUD and available resources	n/a	n/a	n/a	n/a	n/a	n/a	27 (20%)	n/a	**27**
National provider education and patient tracking resources, next steps, and wrap-up^a^	13 (25%)	6 (24%)	24 (13%)	12 (10%)	18 (16%)	12 (16%)	11 (8%)	11 (20%)	**107**
Project overview, site summary, and action plans^a^	7 (14%)	3 (12%)	22 (12%)	27 (22%)	13 (12%)	13 (18%)	13 (9%)	9 (16%)	**107**
Screening and treating OUD with a focus on buprenorphine and naltrexone	12 (24%)	n/a	n/a	12 (10%)	n/a	n/a	n/a	n/a	**24**
Setting up a buprenorphine clinic	12 (24%)	n/a	24 (13%)	27 (22%)	18 (16%)	n/a	n/a	n/a	**81**
What is addiction and how do we treat it?^a^	n/a	7 (28%)	24 (13%)	n/a	n/a	n/a	37 (27%)	n/a	**68**
X-waiver trainings	3 (6%)	5 (20%)	38 (21%)	22 (18%)	15 (14%)	11 (15%)	n/a	5 (9%)	**99**
**Trainings total**	**51**	**25**	**178**	**124**	**110**	**73**	**138**	**56**	
**Interviews**									
Pre-site visit	9 (50%)	7 (50%)	8 (57%)	11 (61%)	7 (58%)	9 (69%)	8 (62%)	9 (75%)	**68**
Post-site visit	9 (50%)	7 (50%)	6 (43%)	7 (39%)	5 (42%)	4 (31%)	5 (38%)	3 (25%)	**46**
**Interviews total**	**18**	**14**	**14**	**18**	**12**	**13**	**13**	**12**	

*n/a* non-requirement and not selected by site

^a^Tailored trainings to sites and time needed to achieve requests may not have been equal nor used identical slides. However, these trainings generally covered the same information

### Implementation phase

The average cost of the implementation phase activities was $17,816 per site and ranged between $10,288 and $30,855 (Table 4, Fig. 2). The average time invested in implementation activities (e.g., trainings, monthly and cross-site facilitation calls) was 260 h per site and ranged from 182 to 380 h (Fig. 2). Similar to the pre-implementation phase, we observed an increase in costs when the hours invested were increased (see Additional file 1: Fig. S1). The index date for implementation was the first day of the site visit and the EF team spent 2 days with local site leaders, clinicians, and staff. There was a lot of heterogeneity in amount of time spent in each implementation phase activity across all sites. For example, Site 3 had the most attendees (*n* = 178) at trainings with 286 total hours contributing to its training costs of $25,064 (Table 5). Conversely, Site 2 had the least attendees (*n* = 25) with 40 total hours contributing to its training costs of $2594. Additionally, Site 3 had the most attendees at X-waiver trainings (*n* = 38) at 152 h which added $12,975 in training costs (Table 5), whereas Site 7 did not spend any time on X-waiver training.

After visiting each site, the external facilitators met separately with LS for monthly facilitation calls and quarterly cross-site collaboration calls for one year. Across all 8 sites, monthly and cross-site calls had 286 and 31 LS encounters, respectively. During the implementation phase, Sites 1 and 2 had the most staff encounters with 63 and 54 documented encounters in the monthly facilitation calls, respectively, and 7 and 10 documented encounters on the cross-site collaboration calls, respectively (Table 5). The number of post-site visit meetings was greater for Sites 1 and 2 because their on-site trainings occurred earlier in the project allowing them more opportunities to join cross-site facilitation calls.

Based on documented hours, external facilitators spent an average of 74 h per site during implementation ranging from 62 and 87 h. The average cost of EF’s effort per site was $3308 (SD 648) (Table 4).

Travel costs were based on two members (A and B) from the EF team who traveled to each site. Costs were grouped by (1) lodging and incidentals and (2) travel (Table 6). Variations in costs were attributed to the time when flight and hotel reservation were booked relative to the visit date and locality rates at different destinations.

**Table 6:** Travel and lodging costs associated with the external facilitators^a^

	Site 1^b^	Site 2^b^	Site 3	Site 4	Site 5	Site 6	Site 7	Site 8	Total costs	Average total costs per site
External facilitator A										
Lodging/incidentals	$308	$308	$426	$466	$449	$559	$1016	$473	$4004	$500
Travel	$424	$424	$643	$788	$626	$836	$493	$447	$4682	$585
**Total**	**$732**	**$732**	**$1069**	**$1254**	**$1075**	**$1395**	**$1509**	**$920**	**$8686**	**$1086**
External facilitator B										
Lodging/incidentals	$318	$318	$384	$437	$385	$532	$716	$1231	$4322	$540
Travel	$268	$268	$701	$734	$1054	$559	$687	$439	$4711	$589
**Total**	**$586**	**$586**	**$1085**	**$1171**	**$1440**	**$1091**	**$1403**	**$1670**	**$9033**	**$1129**
**Totals**	**$1318**	**$1318**	**$2154**	**$2425**	**$2515**	**$2486**	**$2912**	**$2589**	**$17,718**	**$2215**

^a^Costs were collected from the Veterans Health Administration travel and expense management system

^b^Costs were split between sites 1 and 2

### Sensitivity analysis

In the sensitivity analysis using VHA FMS wage table, most of the staff at each of the 8 sites were in the $90 + per hour (average number per site = 15), followed by $60–$90 per hour (average number per site = 9), $30 to $60 per hour (average number per site = 7), less than $30 per hour (average number per site = 0) (see Additional file 1: Table S3). Similarly, when using the BLS wage table, many of the staff at each site were in the $90 or greater per hour wage category (average number per site = 16). Yet variation appeared in other wage categories as many of the staff were in the $30 to $60 per hour (average number per site = 13) category, followed by less than $30 per hour (average number per site = 4), and $60 to $90 per hour (average number per site = 4) categories (Table 3). Wage rates categories showed differences in total costs in the VHA FMS and BLS scales. VHA FMS totals costs were higher in the $90 + ($157,910 vs $122,715) and $60 to $90 categories ($40,010 vs $17,075) compared to BLS wage table. However, BLS totals costs were higher in the lower wage categories of $30 to $60 ($42,489 vs $32,231) and less than $30 categories ($13,780 vs $12,995) compared to the VHA FMS wage table (see Additional file 1: Fig. S2). Additional sensitivity analyses of higher salary costs in the VHA FMS wage table compared to the BLS wage table are presented in Additional file 1: Tables S2, S4, and S5.

## Discussion

This was the first study to measure the facility-level direct costs of implementing an external facilitation intervention for MOUD. Our study reported that implementation costs of an external facilitation program to improve MOUD prescribing at VHA were mainly driven by labor costs. Moreover, these costs were influenced by variations in the time invested at each phase of implementation and the number and type of participants involved with implementing an external facilitation intervention. These findings provide salient accounting information for decision makers who are planning to implement a similar intervention at their medical facilities.

There are few studies that have evaluated the implementation costs of external facilitation. Ritchie and colleagues performed an assessment to capture the costs associated with facilitation of primary care mental health integration (PCMHI) at VHA regional networks [52]. The authors reported that the cost of implementation facilitation varied between $258,127 and $263,490 over a 28-month period. Labor costs of participants, excluding the EF team, comprised of 84 to 88% of the total direct costs. Our study findings were similar in that the labor costs of participants (excluding the EF) comprised 79% of the total implementation costs. However, this varied across sites and ranged from 57 to 90% of the total direct costs. Given that the study by Ritchie and colleagues was done at the regional network level and lasted two years, it was difficult to draw comparisons in terms of costs with our own implementation facilitation. However, the similarity in the proportion of labor that involved participants other than the external facilitation team supports the idea that local participation is a major driver of costs.

Each site had different needs, which are reflected by the heterogeneity of the time invested across the implementation key activities. Some sites required more X-waiver trainings (e.g., Site 3), while others did not invest time into this activity (e.g., Site 7). As more time was invested in each site, total costs increased (see Additional file 1: Fig. S1). These variations highlight the demand for flexible targeting of external facilitation based on the site’s needs, which will impact the costs of implementation. By providing the costs and key activities for each site, we have allowed stakeholders to make an informative decision on implementing an external facilitation program at their centers.

We opted to include the average total implementation cost per site alongside the cost per patient to provide stakeholders with varying perspectives of the cost estimates. Cost analyses such as the budget impact analysis summarize the cost of adopting evidence-based practice at the patient level (e.g., cost per patient, cost per member per month) [53]. This is done to provide a convenient method to scale up the intervention. However, with implementation interventions such as external facilitation, the target is a system-wide effort designed to change the practice culture to impact the patient. Hence, summarizing the overall implementation costs per site makes rational sense given the context of the intervention’s target and goals. Moreover, scalability will depend on the number of patients with OUD; as the number increases, there may be more demand to scale up operations. It is also important to mention that the Drug Enforcement Agency has recently eliminated the need for an X-waiver [54], which could improve access to MOUD patients with OUD. It is unclear how this would impact future costs, but it is an element that stakeholders and decision makers will need to incorporate.

The implementation intervention ended prior to the coronavirus disease 2019 (COVID-19) pandemic and site trainings were held in-person and without travel restrictions. This report has important implications for external facilitation amid travel restrictions due to COVID-19. External facilitation has been performed using both in-person and virtual interactions. However, with the advent of COVID-19 travel restrictions and a move toward more virtual spaces for employment and employee engagement, there could be limitations to the ability to perform in-person external facilitation. Virtual mediums may be a possible alternative to perform such work. Hartmann and colleagues provide guidance on transitioning to a fully virtual external facilitation program, which involves overarching (e.g., pilot testing, adopting a model, prioritizing metacognition) and practical principles (e.g., planning, real-time communication, relationship building, stakeholder engagement, and construction of a virtual room) [55]. However, it’s unclear whether external facilitation functions as well in a virtual space. It may benefit other institutions with limited resources for travel to investigate the effectiveness of virtual versus in-person external facilitation.

While there was no ongoing support from the EF team after the implementation phase, the external facilitators built up local teams across sites during the intervention so local staff could perform maintenance past the intervention. Only the planning and implementation phases were accounted for in the costing timeframe. Identifying the time and cost of labor to sustain an implementation intervention could further help inform policymakers and stakeholders.

Our focus with this assessment was on the costs of implementing an external facilitation intervention. We did not focus on the actual medications that are used for MOUD, such as methadone, buprenorphine, and naltrexone. These pharmaceuticals are low-cost evidence-based interventions that would offset the high costs of opioid use disorder and negative health impacts resulting from OUD. Fairley and colleagues performed a cost-effectiveness analysis to assess the value of using MOUD to treat OUD and reported that MOUDs along with naloxone for harm reduction treatment were cost-effective strategies compared to no treatment [56]. Given that the economic burden of the opioid epidemic exceeded $1 trillion dollars, MOUDs are an affordable evidence-based treatment that should be accessible to patient with OUD [57]. Future studies will need to assess the budget impact of using these MOUDs in a real-world setting.

### Limitations

Despite our best efforts to capture the major domains of implementation costs associated with external facilitation, there were several limitations to this cost analysis. First, it is probable that LS facilitated implementation activities outside of trainings, interviews, and calls with the EF team that were not accounted for in our cost analysis. Since these were not observed or documented, our estimates may be underestimating the actual costs of implementation. Additionally, some external facilitation activities were not included in the accounting. For example, time spent in transit and non-work social functions (e.g., having dinner) with IITs which were used to discuss and overcome implementation barriers. Second, there is a lack of standard frameworks for the cost analysis of implementation science; hence, we approached this using a traditional economic perspective. Attempts to remedy this are underway and several papers have been published to provide guidance on this issue [40, 58]. Meanwhile, we adopted a direct micro-costing approach to capture implementation costs focusing on the planning and implementation phases [59–61]. Micro-costing methods are useful in capturing specific details of activities related to the intervention, but this is dependent on a careful prospective collection of data [42]. Although we did prospectively collect data, multiple participants were needed to collect and input data into our database. Potential for errors and recall bias could impact the overall costs. Alternative methods [62] using the implementation framework by Proctor and colleagues [63] have been developed based on the Time-Driven Activity-Based Costs approach from the business sector [64]. These novel approaches to capturing implementation costs should be considered in future investigations. Next, wage rates for each participant were based on average wage rates from the BLS, which can vary considerably. To address this, we performed a sensitivity analysis using FMS wage rate specific to VHA employees, which resulted in higher implementation costs. This is likely due to the higher wages federal employees incur. According to a systematic review by the Government Accountability Office (GAO-12-564), federal employees made between 4 and 58% more compared to the private sector; however, one study reported that federal employees made 28% less than the private sector [65]. Decision makers will need to validate their wage table to properly account for their labor associated with implementation. Further, we did not capture indirect costs such as the fixed costs of maintaining and operating the facilities. Therefore, our cost estimates are on the lower bound, and decision makers will need to account for the indirect costs when deciding on an implementation plan. Finally, our cost analysis was based in the VHA, which is a large, integrated healthcare system. Stakeholders at VHA may have different incentives compared to non-VHA healthcare systems to implement external facilitation programs, and our findings may not be generalizable to all non-VHA institutions [66]. For example, VHA uses clinical pharmacists differently than other health care systems. While pharmacists fill prescriptions at VHA, they also engage in care management roles [67]. For the current intervention, clinical pharmacists were champions at many of the sites [28, 33]. Moreover, VA does not bill for additional outpatient or ancillary services so the cost benefit to other health care systems might be different. However, there are similarities. This was an external facilitation and the intervention, and the costs associated with it, are applicable to other health care systems. For example, having expert facilitators, champions, and monthly contacts after a site visit can be replicated in other healthcare systems. Thus, large, integrated healthcare systems that are similar to VHA may find our findings informative, particularly if they are planning to implement a similar program at their facility.

## Conclusions

External facilitation may be an affordable strategy to help address the opioid epidemic. Increasing access to MOUD would benefit Veterans through improved health outcomes. Participant composition played an important role in total implementation costs, and decision makers will need to identify the most efficient and optimal number of stakeholders to involve in their implementation plans. However, these implementation costs may be considered a necessary trade-off to address the growing morbidity and mortality of the opioid addiction crisis, and decision makers would benefit from this cost analysis to inform their own implementation plans.

### Supplementary Information

**Additional file 1: Table S1.** Cost per patient, participant, and encounter*. **Table S2.** Number of encounters by external facilitators per facilitation activity. **Table S3.** Number of unique participants by wage category per site^a^ (VHA FMS data). **Table S4.** Costs of local site clinicians, leadership, and staff^a^^,b^ (VA FMS Data). **Table S5.** Costs of external facilitation team (VHA FMS Data). **Figure S1.** Correlation between total hours and total costs for the Planning and Implementation phases. **Figure S2.** Total Costs by Wage Rate Categories^a^.

**Additional file 2.** CHEERS 2022 Checklist.

## Data Availability

The datasets generated and analyzed during the study are not publicly available due to the sensitive nature of cost data but are available from the corresponding author on reasonable request.

## Abbreviations

ADaPT-OUD: Advancing Pharmacological Treatments for Opioid Use Disorder
BLS: Bureau of Labor and Statistics
CPT: Current Procedural Terminology
EF: External facilitation
FMS: Financial Management System
IIT: Internal implementation teams
LS: Local staff
MOUD: Medication treatment for opioid use disorder
OEND: Opioid overdose education and naloxone distribution
OTP: Other training participants
OUD: Opioid use disorder
PCMHI: Primary care mental health integration
SD: Standard deviation
SUD: Substance use disorder
VA: U.S. Department of Veterans Affairs
VHA: Veterans Health Administration
US: United States
