# Supplementary material for: Costs of implementing a multi-site facilitation intervention to increase access to medication treatment for opioid use disorder
Source: Implement Sci Commun. 2023 Aug 10;4:91. doi: 10.1186/s43058-023-00482-8 (PMC10413546; doi:10.1186/s43058-023-00482-8)
Supplement: Supplementary file 1 — Additional file 1: Table S1. Cost per patient, participant, and encounter*. Table S2. Number of encounters by external facilitators per facilitation activity. Table S3. Number of unique participants by wage category per sitea (VHA FMS data). Table S4. Costs of local site clinicians, leadership, and staffa,b (VA FMS Data). Table S5. Costs of external facilitation team (VHA FMS Data). Figure S1. Correlation between total hours and total costs for the Planning and Implementation phases. Figure S2. Total Costs by Wage Rate Categoriesa. [file 43058_2023_482_MOESM1_ESM.zip › Figure A2R1.docx]

**Figure S2**. Total Costs by Wage Rate Categories^a^

^a^Includes 30% overhead costs
