# Supplementary material for: Costs of implementing a multi-site facilitation intervention to increase access to medication treatment for opioid use disorder
Source: Implement Sci Commun. 2023 Aug 10;4:91. doi: 10.1186/s43058-023-00482-8 (PMC10413546; doi:10.1186/s43058-023-00482-8)
Supplement: Supplementary file 1 — Additional file 1: Table S1. Cost per patient, participant, and encounter*. Table S2. Number of encounters by external facilitators per facilitation activity. Table S3. Number of unique participants by wage category per sitea (VHA FMS data). Table S4. Costs of local site clinicians, leadership, and staffa,b (VA FMS Data). Table S5. Costs of external facilitation team (VHA FMS Data). Figure S1. Correlation between total hours and total costs for the Planning and Implementation phases. Figure S2. Total Costs by Wage Rate Categoriesa. [file 43058_2023_482_MOESM1_ESM.zip › Table A1R1.docx]

| **Table S1.** Cost per patient, participant, and encounter.* | | | | | | |
| --- | --- | --- | --- | --- | --- | --- |
|  |  | BLS |  |  | VHA FMS |  |
| Site | Cost per patient | Cost per participant | Cost per encounter | Cost per patient | Cost per participant | Cost per encounter |
| 1 | $164 | $889 | $91 | $204 | $1,106 | $114 |
| 2 | $83 | $613 | $71 | $100 | $745 | $86 |
| 3 | $173 | $721 | $137 | $218 | $910 | $173 |
| 4 | $43 | $650 | $103 | $54 | $804 | $128 |
| 5 | $37 | $722 | $92 | $46 | $892 | $114 |
| 6 | $19 | $674 | $89 | $24 | $842 | $111 |
| 7 | $36 | $523 | $117 | $45 | $656 | $147 |
| 8 | $40 | $630 | $98 | $50 | $784 | $122 |
| Average total costs per site^†^ | $48 | $664 | $102 | $60 | $828 | $127 |

* Costs represent the wage rate + 30% fringe.

† Average total costs were estimated by taking the total costs divided by the total number of patients, participants, and encounters.

BLS, Bureau of Labor and Statistics

VHA FMS, Veterans Health Administration Financial Management System

*Sensitivity analysis*

Sensitivity analysis was performed to investigate if there were differences in total costs when using different salary scales (BLS and VHA FMS). In the base-case analysis using the BLS salary scale, the average total cost of implementation per site was $18,847 (SD 8,579) (**Table 1**). However, when using the VHA FMS salary scale, the average total cost of implementation per site was $23,475 (SD, $8,579). Similar findings were reported when including the 30% fringe benefits to the wage rate for the BLS and VHA FMS salary scales ($24,501 and $30,518, respectively). Differences in salary scales were noted in the wage rate categories.
