# Supplementary material for: Costs of implementing a multi-site facilitation intervention to increase access to medication treatment for opioid use disorder
Source: Implement Sci Commun. 2023 Aug 10;4:91. doi: 10.1186/s43058-023-00482-8 (PMC10413546; doi:10.1186/s43058-023-00482-8)
Supplement: Supplementary file 1 — Additional file 1: Table S1. Cost per patient, participant, and encounter*. Table S2. Number of encounters by external facilitators per facilitation activity. Table S3. Number of unique participants by wage category per sitea (VHA FMS data). Table S4. Costs of local site clinicians, leadership, and staffa,b (VA FMS Data). Table S5. Costs of external facilitation team (VHA FMS Data). Figure S1. Correlation between total hours and total costs for the Planning and Implementation phases. Figure S2. Total Costs by Wage Rate Categoriesa. [file 43058_2023_482_MOESM1_ESM.zip › Table A2R1.docx]

**Table S2.** Number of encounters by external facilitators per facilitation activity

|  | Facilitation Activities | External Facilitator  A | External Facilitator  B | External Facilitator  C | External Facilitator  D | External Facilitator  E | External Facilitator  F | External Facilitator G | External Facilitator H |
| --- | --- | --- | --- | --- | --- | --- | --- | --- | --- |
| Site 1 |  |  |  |  |  |  |  |  |  |
|  | Monthly Facilitation Call | 10 (36%) | 4 (19%) | 11 (21%) | 0 (0%) | n/a | n/a | 1 (17%) | 0 (0%) |
|  | Cross-Site Calls | 3 (11%) | 1 (5%) | 3 (6%) | 0 (0%) | n/a | n/a | 2 (33%) | 1 (100%) |
|  | Facilitation Training^a^ | 6^a^ (21%) | 6 (29%) | 0 (0%) | 0 (0%) | n/a | n/a | 0 (0%) | 0 (0%) |
|  | Pre-implementation Interview | 3 (11%) | 0 (0%) | 4 (8%) | 3 (50%) | n/a | n/a | 0 (0%) | 0 (0%) |
|  | Post-implementation Interview | 0 (0%) | 0 (0%) | 3 (6%) | 3 (50%) | n/a | n/a | 3 (50%) | 0 (0%) |
|  | Education | 0 (0%) | 3 (14%) | 0 (0%) | 0 (0%) | n/a | n/a | 0 (0%) | 0 (0%) |
|  | Network Development | 0 (0%) | 2 (10%) | 0 (0%) | 0 (0%) | n/a | n/a | 0 (0%) | 0 (0%) |
|  | Prep/Planning for Site Visit | 4 (14%) | 3 (14%) | 1 (2%) | 0 (0%) | n/a | n/a | 0 (0%) | 0 (0%) |
|  | Problem Identification | 2 (7%) | 0 (0%) | 0 (0%) | 0 (0%) | n/a | n/a | 0 (0%) | 0 (0%) |
|  | Other | 0 (0%) | 2 (10%) | 0 (0%) | 0 (0%) | n/a | n/a | 0 (0%) | 0 (0%) |
|  | Implementation Administration | 0 (0%) | 0 (0%) | 30 (58%) | 0 (0%) | n/a | n/a | 0 (0%) | 0 (0%) |
|  |  |  |  |  |  |  |  |  |  |
| Site 2 |  |  |  |  |  |  |  |  |  |
|  | Monthly Facilitation Call | 12 (34%) | 3 (16%) | 12 (22%) | 0 (0%) | 0 (0%) | n/a | 0 (0%) | n/a |
|  | Cross-Site Calls | 2 (6%) | 2 (11%) | 1 (2%) | 0 (0%) | 1 (20%) | n/a | 22 (92%) | n/a |
|  | Facilitation Training^a^ | 6^a^ (17%) | 6 (32%) | 0 (0%) | 0 (0%) | 0 (0%) | n/a | 0 (0%) | n/a |
|  | Pre-implementation Interview | 3 (9%) | 0 (0%) | 2 (4%) | 2 (50%) | 2 (40%) | n/a | 0 (0%) | n/a |
|  | Post-implementation Interview | 0 (0%) | 0 (0%) | 3 (6%) | 2 (50%) | 2 (40%) | n/a | 2 (8%) | n/a |
|  | Assessment | 1 (3%) | 0 (0%) | 0 (0%) | 0 (0%) | 0 (0%) | n/a | 0 (0%) | n/a |
|  | Education | 0 (0%) | 4 (21%) | 0 (0%) | 0 (0%) | 0 (0%) | n/a | 0 (0%) | n/a |
|  | Network Development | 1 (3%) | 2 (11%) | 0 (0%) | 0 (0%) | 0 (0%) | n/a | 0 (0%) | n/a |
|  | Prep/Planning for Site Visit | 4 (11%) | 2 (11%) | 0 (0%) | 0 (0%) | 0 (0%) | n/a | 0 (0%) | n/a |
|  | Problem Identification | 3 (9%) | 0 (0%) | 0 (0%) | 0 (0%) | 0 (0%) | n/a | 0 (0%) | n/a |
|  | Process Monitoring | 1 (3%) | 0 (0%) | 0 (0%) | 0 (0%) | 0 (0%) | n/a | 0 (0%) | n/a |
|  | Stakeholder Engagement | 1 (3%) | 0 (0%) | 0 (0%) | 0 (0%) | 0 (0%) | n/a | 0 (0%) | n/a |
|  | Other | 1 (3%) | 0 (0%) | 0 (0%) | 0 (0%) | 0 (0%) | n/a | 0 (0%) | n/a |
|  | Implementation Administration | 0 (0%) | 0 (0%) | 36 (67%) | 0 (0%) | 0 (0%) | n/a | 0 (0%) | n/a |
|  |  |  |  |  |  |  |  |  |  |
| Site 3 |  |  |  |  |  |  |  |  |  |
|  | Monthly Facilitation Call | 4 (19%) | 1 (8%) | 8 (18%) | n/a | n/a | 0 (0%) | 0 (0%) | 0 (0%) |
|  | Cross-Site Calls | 1 (5%) | 1 (8%) | 1 (2%) | n/a | n/a | 0 (0%) | 0 (0%) | 1 (100%) |
|  | Facilitation Training^a^ | 6^a^ (29%) | 7 (54%) | 0 (0%) | n/a | n/a | 0 (0%) | 0 (0%) | 0 (0%) |
|  | Pre-implementation Interview | 3 (14%) | 0 (0%) | 3 (7%) | n/a | n/a | 1 (100%) | 0 (0%) | 0 (0%) |
|  | Post-implementation Interview | 0 (0%) | 0 (0%) | 5 (11%) | n/a | n/a | 0 (0%) | 1 (100%) | 0 (0%) |
|  | Education | 0 (0%) | 3 (23%) | 0 (0%) | n/a | n/a | 0 (0%) | 0 (0%) | 0 (0%) |
|  | Prep/Planning for Site Visit | 3 (14%) | 1 (8%) | 0 (0%) | n/a | n/a | 0 (0%) | 0 (0%) | 0 (0%) |
|  | Problem Identification | 3 (14%) | 0 (0%) | 0 (0%) | n/a | n/a | 0 (0%) | 0 (0%) | 0 (0%) |
|  | Process Monitoring | 0 (0%) | 0 (0%) | 0 (0%) | n/a | n/a | 0 (0%) | 0 (0%) | 0 (0%) |
|  | Implementation Administration | 0 (0%) | 0 (0%) | 27 (61%) | n/a | n/a | 0 (0%) | 0 (0%) | 0 (0%) |
|  | Other | 1 (5%) | 0 (0%) | 0 (0%) | n/a | n/a | 0 (0%) | 0 (0%) | 0 (0%) |
| Site 4 |  |  |  |  |  |  |  |  |  |
|  | Monthly Facilitation Call | 8 (29%) | 3 (23%) | 10 (20%) | 0 (0%) | n/a | 0 (0%) | 2 (40%) | 0 (0%) |
|  | Cross-Site Calls | 1 (4%) | 1 (8%) | 1 (2%) | 0 (0%) | n/a | 0 (0%) | 1 (20%) | 1 (100%) |
|  | Facilitation Training^a^ | 6^a^ (21%) | 7 (54%) | 0 (0%) | 0 (0%) | n/a | 0 (0%) | 0 (0%) | 0 (0%) |
|  | Pre-implementation Interview | 3 (11%) | 0 (0%) | 5 (10%) | 2 (50%) | n/a | 3 (100%) | 0 (0%) | 0 (0%) |
|  | Post-implementation Interview | 0 (0%) | 0 (0%) | 3 (6%) | 2 (50%) | n/a | 0 (0%) | 2 (40%) | 0 (0%) |
|  | Education | 1 (4%) | 1 (8%) | 0 (0%) | 0 (0%) | n/a | 0 (0%) | 0 (0%) | 0 (0%) |
|  | Prep/Planning for Site Visit | 1 (4%) | 1 (8%) | 0 (0%) | 0 (0%) | n/a | 0 (0%) | 0 (0%) | 0 (0%) |
|  | Problem Identification/Solving | 4 (14%) | 0 (0%) | 0 (0%) | 0 (0%) | n/a | 0 (0%) | 0 (0%) | 0 (0%) |
|  | Program Marketing | 1 (4%) | 0 (0%) | 0 (0%) | 0 (0%) | n/a | 0 (0%) | 0 (0%) | 0 (0%) |
|  | Implementation Administration | 0 (0%) | 0 (0%) | 31 (62%) | 0 (0%) | n/a | 0 (0%) | 0 (0%) | 0 (0%) |
|  | Other | 3 (11%) | 0 (0%) | 0 (0%) | 0 (0%) | n/a | 0 (0%) | 0 (0%) | 0 (0%) |
|  |  |  |  |  |  |  |  |  |  |
| Site 5 |  |  |  |  |  |  |  |  |  |
|  | Monthly Facilitation Call | 9 (45%) | 1 (9%) | 7 (16%) | 0 (0%) | 0 (0%) | n/a | 8 (67%) | 0 (0%) |
|  | Cross-Site Calls | 2 (10%) | 2 (18%) | 1 (2%) | 0 (0%) | 1 (100%) | n/a | 2 (17%) | 1 (100%) |
|  | Facilitation Training^a^ | 6^a^ (30%) | 6 (55%) | 0 (0%) | 0 (0%) | 0 (0%) | n/a | 0 (0%) | 0 (0%) |
|  | Pre-implementation Interview | 1 (5%) | 0 (0%) | 4 (9%) | 3 (100%) | 0 (0%) | n/a | 0 (0%) | 0 (0%) |
|  | Post-implementation Interview | 0 (0%) | 0 (0%) | 3 (7%) | 0 (0%) | 0 (0%) | n/a | 2 (17%) | 0 (0%) |
|  | Education | 0 (0%) | 1 (9%) | 0 (0%) | 0 (0%) | 0 (0%) | n/a | 0 (0%) | 0 (0%) |
|  | Prep/Planning for Site Visit | 2 (10%) | 1 (9%) | 0 (0%) | 0 (0%) | 0 (0%) | n/a | 0 (0%) | 0 (0%) |
|  | Implementation Administration | 0 (0%) | 0 (0%) | 29 (66%) | 0 (0%) | 0 (0%) | n/a | 0 (0%) | 0 (0%) |
|  |  |  |  |  |  |  |  |  |  |
| Site 6 |  |  |  |  |  |  |  |  |  |
|  | Monthly Facilitation Call | 10 (40%) | 0 (0%) | 10 (25%) | 0 (0%) | 0 (0%) | n/a | 2 (67%) | 0 (0%) |
|  | Cross-Site Calls | 1 (4%) | 1 (8%) | 1 (3%) | 0 (0%) | 0 (0%) | n/a | 1 (33%) | 1 (100%) |
|  | Facilitation Training^a^ | 6^a^ (24%) | 7 (58%) | 0 (0%) | 0 (0%) | 0 (0%) | n/a | 0 (0%) | 0 (0%) |
|  | Pre-implementation Interview | 3 (12%) | 0 (0%) | 5 (13%) | 2 (67%) | 0 (0%) | n/a | 0 (0%) | 0 (0%) |
|  | Post-implementation Interview | 1 (4%) | 0 (0%) | 1 (3%) | 1 (33%) | 1 (100%) | n/a | 0 (0%) | 0 (0%) |
|  | Education | 0 (0%) | 2 (17%) | 0 (0%) | 0 (0%) | 0 (0%) | n/a | 0 (0%) | 0 (0%) |
|  | Network Development | 0 (0%) | 1 (8%) | 0 (0%) | 0 (0%) | 0 (0%) | n/a | 0 (0%) | 0 (0%) |
|  | Prep/Planning for Site Visit | 2 (8%) | 1 (8%) | 0 (0%) | 0 (0%) | 0 (0%) | n/a | 0 (0%) | 0 (0%) |
|  | Problem Identification/Solving | 1 (4%) | 0 (0%) | 0 (0%) | 0 (0%) | 0 (0%) | n/a | 0 (0%) | 0 (0%) |
|  | Implementation Administration | 0 (0%) | 0 (0%) | 23 (58%) | 0 (0%) | 0 (0%) | n/a | 0 (0%) | 0 (0%) |
|  | Other | 1 (4%) | 0 (0%) | 0 (0%) | 0 (0%) | 0 (0%) | n/a | 0 (0%) | 0 (0%) |
|  |  |  |  |  |  |  |  |  |  |
| Site 7 |  |  |  |  |  |  |  |  |  |
|  | Monthly Facilitation Call | 8 (40%) | 0 (0%) | 12 (27%) | 0 (0%) | 1 (50%) | n/a | 4 (57%) | n/a |
|  | Cross-Site Calls | 1 (5%) | 1 (11%) | 0 (0%) | 0 (0%) | 1 (50%) | n/a | 1 (14%) | n/a |
|  | Facilitation Training^a^ | 7^a^ (35%) | 7 (78%) | 0 (0%) | 0 (0%) | 0 (0%) | n/a | 0 (0%) | n/a |
|  | Pre-implementation Interview | 3 (15%) | 0 (0%) | 3 (7%) | 2 (67%) | 0 (0%) | n/a | 2 (29%) | n/a |
|  | Post-implementation Interview | 0 (0%) | 0 (0%) | 2 (4%) | 1 (33%) | 0 (0%) | n/a | 0 (0%) | n/a |
|  | Prep/Planning for Site Visit | 1 (5%) | 1 (11%) | 0 (0%) | 0 (0%) | 0 (0%) | n/a | 0 (0%) | n/a |
|  | Implementation Administration | 0 (0%) | 0 (0%) | 28 (62%) | 0 (0%) | 0 (0%) | n/a | 0 (0%) | n/a |
|  |  |  |  |  |  |  |  |  |  |
| Site 8 |  |  |  |  |  |  |  |  |  |
|  | Monthly Facilitation Call | 8 (40%) | 0 (0%) | 9 (21%) | 0 (0%) | n/a (%) | n/a | 0 (0%) | 0 (0%) |
|  | Cross-Site Calls | 1 (5%) | 1 (11%) | 1 (2%) | 0 (0%) | n/a (%) | n/a | 1 (100%) | 1 (100%) |
|  | Facilitation Training^a^ | 5^a^ (25%) | 6 (67%) | 0 (0%) | 0 (0%) | n/a (%) | n/a | 0 (0%) | 0 (0%) |
|  | Pre-implementation Interview | 4 (20%) | 0 (0%) | 5 (12%) | 3 (60%) | n/a (%) | n/a | 0 (0%) | 0 (0%) |
|  | Post-implementation Interview | 0 (0%) | 0 (0%) | 0 (0%) | 2 (40%) | n/a (%) | n/a | 0 (0%) | 0 (0%) |
|  | Education | 0 (0%) | 1 (11%) | 0 (0%) | 0 (0%) | n/a (%) | n/a | 0 (0%) | 0 (0%) |
|  | Prep/Planning for Site Visit | 1 (5%) | 1 (11%) | 0 (0%) | 0 (0%) | n/a (%) | n/a | 0 (0%) | 0 (0%) |
|  | Program Marketing | 1 (5%) | 0 (0%) | 0 (0%) | 0 (0%) | n/a (%) | n/a | 0 (0%) | 0 (0%) |
|  | Implementation Administration | 0 (0%) | 0 (0%) | 27 (64%) | 0 (0%) | n/a (%) | n/a | 0 (0%) | 0 (0%) |

^a^Includes facilitator’s time when they were an observer at on-site trainings
