# Supplementary material for: Costs of implementing a multi-site facilitation intervention to increase access to medication treatment for opioid use disorder
Source: Implement Sci Commun. 2023 Aug 10;4:91. doi: 10.1186/s43058-023-00482-8 (PMC10413546; doi:10.1186/s43058-023-00482-8)
Supplement: Supplementary file 1 — Additional file 1: Table S1. Cost per patient, participant, and encounter*. Table S2. Number of encounters by external facilitators per facilitation activity. Table S3. Number of unique participants by wage category per sitea (VHA FMS data). Table S4. Costs of local site clinicians, leadership, and staffa,b (VA FMS Data). Table S5. Costs of external facilitation team (VHA FMS Data). Figure S1. Correlation between total hours and total costs for the Planning and Implementation phases. Figure S2. Total Costs by Wage Rate Categoriesa. [file 43058_2023_482_MOESM1_ESM.zip › Table A5_R1.docx]

| **Table S5.** Costs of external facilitation team (VHA FMS Data) | | | | | | | | | | |  |  |  |  |  |  |  |
| --- | --- | --- | --- | --- | --- | --- | --- | --- | --- | --- | --- | --- | --- | --- | --- | --- | --- |
|  | Planning phase^a^ | | | | Implementation phase^a^ | | | | Total | | | Average and Standard Deviations | | | | | |
|  | Hours | Cost | Cost times 30% overhead | Days in phase | Hours | Cost | Cost  times 30% overhead | Days in phase | Hours | Total Cost | Total Cost times 30% overhead | Hours | SD | Average Total Costs | SD | Average Total Costs + 30% overhead | SD |
| Site 1^b^ |  |  |  |  |  |  |  |  |  |  |  |  |  |  |  |  |  |
| External Facilitation Team | 17 | $1,031 | $1,340 | 60 days | 83 | $5,236 | $6,807 | 783 days | 100 | $6,267 | $8,147 | 50 | 46 | $3,134 | $2,973 | $4,074 | $3,865 |
|  |  |  |  |  |  |  |  |  |  |  |  |  |  |  |  |  |  |
| Site 2^b^ |  |  |  |  |  |  |  |  |  |  |  |  |  |  |  |  |  |
| External Facilitation Team | 16 | $806 | $1,048 | 54 days | 87 | $5,030 | $6,539 | 785 days | 103 | $5,836 | $7,587 | 52 | 50 | $2,918 | $2,987 | $3,793 | $3,883 |
|  |  |  |  |  |  |  |  |  |  |  |  |  |  |  |  |  |  |
| Site 3 |  |  |  |  |  |  |  |  |  |  |  |  |  |  |  |  |  |
| External Facilitation Team | 8 | $623 | $810 | 153 days | 65 | $3,552 | $4,618 | 666 days | 73 | $4,175 | $5,428 | 36 | 40 | $2,088 | $2,071 | $2,714 | $2,692 |
|  |  |  |  |  |  |  |  |  |  |  |  |  |  |  |  |  |  |
| Site 4 |  |  |  |  |  |  |  |  |  |  |  |  |  |  |  |  |  |
| External Facilitation Team | 11 | $680 | $884 | 160 days | 82 | $4,319 | $5,615 | 659 days | 92 | $4,999 | $6,499 | 47 | 50 | $2,500 | $2,573 | $3,249 | $3,345 |
|  |  |  |  |  |  |  |  |  |  |  |  |  |  |  |  |  |  |
| Site 5 |  |  |  |  |  |  |  |  |  |  |  |  |  |  |  |  |  |
| External Facilitation Team | 15 | $564 | $733 | 259 days | 66 | $3,451 | $4,486 | 560 days | 81 | $4,015 | $5,220 | 41 | 36 | $2,008 | $2,041 | $2,610 | $2,654 |
|  |  |  |  |  |  |  |  |  |  |  |  |  |  |  |  |  |  |
| Site 6 |  |  |  |  |  |  |  |  |  |  |  |  |  |  |  |  |  |
| External Facilitation Team | 12 | $631 | $820 | 504 days | 62 | $3,178 | $4,131 | 365 days | 72 | $3,809 | $4,952 | 37 | 35 | $1,905 | $1,801 | $2,476 | $2,341 |
|  |  |  |  |  |  |  |  |  |  |  |  |  |  |  |  |  |  |
| Site 7 |  |  |  |  |  |  |  |  |  |  |  |  |  |  |  |  |  |
| External Facilitation Team | 12 | $832 | $1,082 | 357 days | 74 | $3,813 | $4,957 | 365 days | 87 | $4,645 | $6,039 | 43 | 44 | $2,323 | $2,108 | $3,019 | $2,740 |
|  |  |  |  |  |  |  |  |  |  |  |  |  |  |  |  |  |  |
| Site 8 |  |  |  |  |  |  |  |  |  |  |  |  |  |  |  |  |  |
| External Facilitation Team | 9 | $667 | $867 | 364 days | 71 | $3,569 | $4,640 | 365 days | 80 | $4,236 | $5,507 | 40 | 44 | $2,118 | $2,052 | $2,753 | $2,668 |
|  |  |  |  |  |  |  |  |  |  |  |  |  |  |  |  |  |  |
| **Total hours and costs** | **100** | **$5,834** | **$7,584** |  | **589** | **$32,148** | **$41,792** |  | **688** | **$37,982** | **$49,377** | **345** | **346** | **$18,991** | **$18,607** | **$24,688** | **$24,189** |

^a^Some activities' (e.g., work on SharePoint site, REDCap, drafting site resources) time and costs were distributed evenly across all sites.

^b^Site 1 and 2 had consecutive site visit dates so some administrative time and costs were split among these sites
